# Antibacterial Compounds from Marine *Vibrionaceae* Isolated on a Global Expedition

**DOI:** 10.3390/md8122946

**Published:** 2010-12-13

**Authors:** Matthias Wietz, Maria Mansson, Charlotte H. Gotfredsen, Thomas O. Larsen, Lone Gram

**Affiliations:** 1 National Food Institute, Technical University of Denmark, 2800 Kgs. Lyngby, Denmark; E-Mail: mwie@food.dtu.dk; 2 Centre for Microbial Biotechnology, Department of Systems Biology, Technical University of Denmark, 2800 Kgs. Lyngby, Denmark; E-Mails: maj@bio.dtu.dk (M.M.); tol@bio.dtu.dk (T.O.L.); 3 Department of Chemistry, Technical University of Denmark, 2800 Kgs. Lyngby, Denmark; E-Mail: chg@kemi.dtu.dk

**Keywords:** *Vibrio coralliilyticus*, *Vibrio neptunius*, *Photobacterium halotolerans*, chemotyping, andrimid, holomycin

## Abstract

On a global research expedition, over 500 bacterial strains inhibitory towards pathogenic bacteria were isolated. Three hundred of the antibacterial strains were assigned to the *Vibrionaceae* family. The purpose of the present study was to investigate the phylogeny and bioactivity of five *Vibrionaceae* strains with pronounced antibacterial activity. These were identified as *Vibrio coralliilyticus* (two strains), *V. neptunius* (two strains), and *Photobacterium halotolerans* (one strain) on the basis of housekeeping gene sequences. The two related *V. coralliilyticus* and *V. neptunius* strains were isolated from distant oceanic regions. Chemotyping by LC-UV/MS underlined genetic relationships by showing highly similar metabolite profiles for each of the two *V. coralliilyticus* and *V. neptunius* strains, respectively, but a unique profile for *P. halotolerans*. Bioassay-guided fractionation identified two known antibiotics as being responsible for the antibacterial activity; andrimid (from *V. coralliilyticus*) and holomycin (from *P. halotolerans*). Despite the isolation of already known antibiotics, our findings show that marine *Vibrionaceae* are a resource of antibacterial compounds and may have potential for future natural product discovery.

## 1. Introduction

Bioactive secondary metabolites are believed to play a key role in microbial interactions by mediating antagonistic activity and intercellular communication [1]. In addition, many microbial natural products have biotechnological potential as antibiotics, biosurfactants, antifungal, or anticancer agents [2]. Sequences of microbial genomes revealed that only a small fraction of the natural product diversity is known, highlighting the potential for finding novel bioactive compounds in environmental microorganisms [3]. The need for novel antimicrobials to combat increasing antibiotic resistances in pathogenic bacteria has stimulated the exploration of other than the traditional sources, such as terrestrial actinomycetes or fungi [4].

The marine environment harbors bacteria with antagonistic traits [5,6], and marine microorganisms are a potential source of novel antimicrobials [7]. Antagonistic marine bacteria have been isolated from surface [8] and deep waters [9], but the majority originated from biotic surfaces such as sponges [10], zooplankton and macroalgae [8,11], corals [12], and bryozoans [13]. Bioactive bacterial strains predominantly belong to *Pseudoalteromonas* spp. [14], the *Roseobacter* clade [15], and *Actinobacteria* [16]. A number of marine-derived antimicrobials have been characterized in greater detail, including halogenated [17] and sulfuric [18] compounds, depsipeptides [19] and lipopeptides [20], glycolipids [21], as well as high molecular weight structures such as amino acid oxidases [22].

Also the *Vibrionaceae* family, Gram-negative *Gammaproteobacteria* ubiquitous in marine and brackish environments [23], harbors strains with antagonistic activity [8]. The family comprises eight genera, with *Vibrio* and *Photobacterium* constituting the majority of species. To date, *Vibrionaceae* have primarily been investigated due to their pathogenic potential to humans and aquatic animals, but they also occur in commensal or symbiotic associations with eukaryotic organisms [23]. While the abundance of *Vibrionaceae* in nutrient-rich microenvironments such as chitinous zooplankton is potentially related to a superior nutrient utilization based on their metabolic versatility [24], antagonism of competing bacteria through production of antimicrobial compounds may also contribute to a selective advantage. Antimicrobials from *Vibrio* spp. can reduce the number of other microbial community members and influence microscale variations in competing bacterial populations [6]. Antibacterial activities have been described from *V. alginolyticus* [25], *V. parahaemolyticus* [26], *V. anguillarum* [27], and several unidentified *Vibrio* spp. [28,29]. However, the nature and frequency of antagonism among vibrios is still largely unknown, and only a few antibiotic *Vibrio* compounds have been structure elucidated to date [30,31].

The present study describes the analysis of bioactive *Vibrionaceae* strains collected during a global marine expedition [8]. The purpose was to (i) provide phylogenetic and chemical analyses of the strains with strongest antibacterial activity; (ii) characterize their bioactivity depending on culture conditions; and (iii) isolate and elucidate the structure of bioactive metabolites. We report the identification of five *Vibrionaceae* strains with pronounced antibacterial activity, the use of chemotyping to support genetic identification, and the structures of two antibacterial compounds.

## 2. Results and Discussion

### 2.1. Selection of Strains with Pronounced Antibacterial Activity

**Figure 1:**
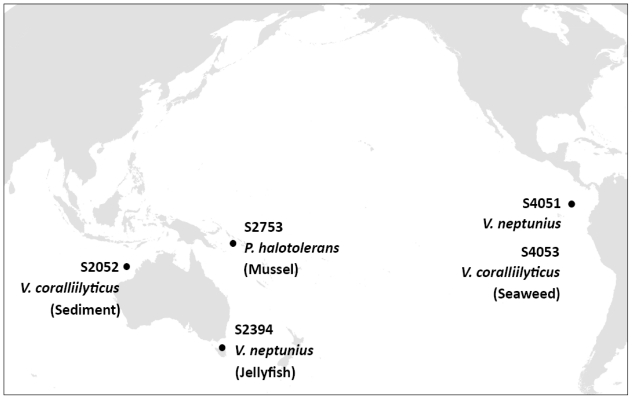
Site of isolation, source, and species identification of five bioactive marine *Vibrionaceae*. Strains were identified to the species level by sequence analysis of several housekeeping genes (see below).

Three hundred and one *Vibrionaceae* strains were isolated during a global marine expedition (http://www.galathea3.dk/uk) based on their ability to antagonize the fish pathogen *Vibrio anguillarum* strain 90-11-287 [8]. After being stored at −80 °C for between six and 12 months, all strains were retested for antibacterial activity against *V. anguillarum* strain 90-11-287 and the human pathogen *Staphylococcus aureus* strain 8325 by spotting colony mass on pathogen-seeded agar [8]. Activity was assessed by the formation of clearing zones around spotted colony mass. From 301 strains, only 138 retained antibacterial activity, being a small fraction compared to other antagonistic marine bacteria [32,33]. One hundred strains causing pronounced inhibition (diameter of clearing zones larger than 10 mm) were retested using the same set-up, resulting in a subselection of 39 strains with reproducible strong antibacterial activity when spotted on pathogen-seeded agar. This subselection was inoculated in liquid cultures and extracted with ethyl acetate to determine if antibacterial compounds were extractable with organic solvent. Activity was seen in ethyl acetate extracts from five strains, which were selected for further analyses. The five bioactive strains originated from different surface samples collected in distant oceanic regions (Figure 1).

### 2.2. Phylogenetic Identification and Chemotyping of Strains

**Figure 2:**
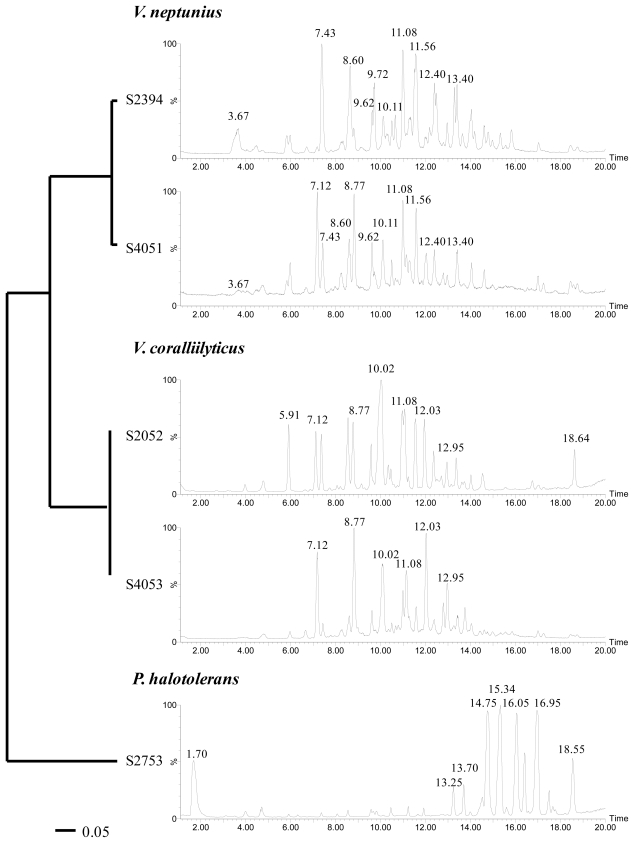
Phylogenetic and chemical relationship between five bioactive *Vibrionaceae* based on neighbor-joining analyses of aligned *toxR* gene sequences and LC-MS Total Ion Chromatograms (TIC). The scale bar relates to the number of base substitutions in *toxR* gene sequences (as displayed by branch lengths in the phylogenetic tree).

All strains investigated in the present study had previously been assigned to the *Vibrionaceae* family based on 16S rRNA gene similarities [8]. However, the 16S rRNA gene is highly conserved among the *Vibrionaceae* and is not well suited for identification to the species level [34]. Therefore, additional sequence analyses of three housekeeping genes (*recA*, *rpoA*, and *toxR*) were performed. These genes encode constitutively expressed proteins and are suitable for phylogenetic studies of *Vibrionaceae* [34,35]. On the basis of *recA* and *rpoA* sequence similarities, strains S2052 and S4053 were identified as *Vibrio coralliilyticus*, S2394 and S4051 as *Vibrio neptunius*, and S2753 as *Photobacterium halotolerans* (Figure 1). The *toxR* gene was less suited for general species identification due to its high variability even in closely related vibrios, as well as comparatively few *toxR* sequence data available in public gene libraries [36]. However, multiple alignments and neighbor-joining analyses of *toxR* sequences provided the best phylogenetic resolution for determining the relationship between the five strains (Figure 2). The usefulness of *toxR* for species discrimination was consistent with previous reports [35]. LC-UV/MS metabolite profiles underlined the close relationship between *V. coralliilyticus* S2052/4053 and *V. neptunius* S2394/4051, respectively. The evolutionary distance of *P. halotolerans* S2753 to the other strains was reflected by a unique metabolite profile (Figure 2). All five strains were consistent in their metabolite production in separate cultivations over a one-year interval.

Several metabolites were produced by all *V. coralliilyticus* and *V. neptunius* strains, for instance those related to the peaks at retention times Rt = 11.08 and 12.03 min (Figure 2). Although they are different species, *V. coralliilyticus* and *V. neptunius* are closely related vibrios with only 2–3% sequence variation in the *recA* and *rpoA* genes (data not shown), signifying why biosynthetic pathways are shared between the species. Based on their molecular formulas, UV, and MS characteristics [37], most of the metabolites produced by both species were assigned as smaller peptides (*m/z* 300–500), a class of molecules commonly produced by marine culturable bacteria [38,39]. Despite the presence of shared metabolites between *V. coralliilyticus* and *V. neptunius*, clearly distinguishable peaks were seen as well. For instance, the major peak at retention time Rt = 10.02 min (MW 479 Da) was only seen in the two *V. coralliilyticus*, and the peak at Rt = 10.11 min (MW 493 Da) only in the two *V. neptunius* strains.

The metabolites produced by *P. halotolerans* S2753 comprised a series of larger peptides (*m/z* 500–900) [40]. The large peak at Rt = 1.70 min (MW 213 Da) displayed a unique UV spectrum characteristic of that of a highly conjugated system. However, this peak could not be ascribed to any known compound or compound class based on LC-UV/MS data alone.

Several metabolites (Rt = 4.70, 7.41, 8.60, 9.60, and 10.50 min) were found in all five strains and assigned as poly-β-hydroxybutyric acid polymers (PHB) of varying lengths (repeating unit n = 86 Da). This was verified by NMR for some of the compounds (data not shown). PHB are common bacterial storage compounds accumulated when growing on an excess carbon source [41].

Chemotyping of prokaryotes has mostly been restricted to analyses of fatty acids and sugars [42], but we show that also the profiling of small molecules can be used for species discrimination. This highlights the usefulness of metabolomics for bacterial classification, adding to recent work of whole-cell laser desorption MALDI-TOF mass spectrometry for characterization of vibrios [43] and secondary metabolite profiling to assess the biosynthetic potential of marine *Pseudoalteromonas* [32]. While our study is limited to the analysis of only three species from the *Vibrionaceae* family, the isolation of two genetically and chemically closely related “strain siblings” from distant oceanic regions indicated that production of certain secondary metabolites is a preserved trait. Similar secondary metabolite profiles were also shown for marine actinomycetal *Salinispora* spp. [44] from distant habitats. Also, all *Ruegeria mobilis* strains from worldwide locations produced the same antibiotic, tropodithietic acid [33].

### 2.3. Bioassay-Guided Identification of Antibacterial Compounds

*V. coralliilyticus* (strains S2052 and S4053) and *P. halotolerans* (S2753) inhibited both *V. anguillarum* and *S. aureus*, whereas *V. neptunius* (strains S2394 and S4051) only inhibited *V. anguillarum* (Table 1). Antibacterial activity was highest in aerated cultures and detected after one, three, and five days of incubation. No significant difference in activity was seen between the tested culture media.

The finding of bioactivity among marine *Vibrionaceae* underlined marine microorganisms being a source of antimicrobials. To our knowledge, none of the species investigated here have previously been studied with respect to their secondary metabolome including antibacterial compounds.

To identify the compounds responsible for the observed activity, large-scale cultivations and fractionations were undertaken for *V. coralliilyticus* S2052 and *P. halotolerans* S2753, representing two distant *Vibrionaceae* species with different metabolite profiles. All fractionation steps were guided by activity testing against *V. anguillarum* strain 90-11-287.

**Figure 3:**
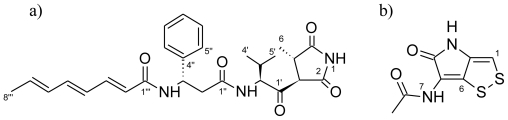
Structures of andrimid (**a**) and holomycin (**b**) isolated from marine *Vibrionaceae*.

Initial dereplication of S2052 by LC-UV/MS [37] and explorative solid-phase extraction (E-SPE) [45] indicated that andrimid (Rt = 10.02 min; Figure 2) could be responsible for the antibacterial activity. This compound (Figure 3a), a hybrid nonribosomal peptide-polyketide antibiotic, was first described from an insect endosymbiont [46] and later found in other microbial species [47,48] including marine vibrios [29,31]. Pure andrimid was isolated for NMR analysis, and our data was in accordance with literature data [47]. Andrimid acts as an acetyl-CoA carboxylase inhibitor [49], and we extended its broad antibiotic spectrum [50] by showing inhibition of the bacterial pathogens *Salmonella* Enteritidis, *Bacillus cereus*, *Yersinia enterolitica*, *Yersinia ruckeri*, *Vibrio harveyi*, and *Vibrio vulnificus* (data not shown). Production of andrimid was also confirmed for the other isolated *V. coralliilyticus* strain, S4053. We furthermore speculate whether a recent report of antagonism in the *V. coralliilyticus* type strain [12] was also attributed to this compound. Previous studies have revealed almost identical andrimid gene clusters and a transposase pseudogene in two producer species, suggesting horizontal gene transfer as the most likely explanation behind the cosmopolitanism of the antibiotic [51]. We hypothesize that such transfer is also the reason for its presence in *V. coralliilyticus* S2052 and S4053. Our study is the first linking andrimid production to a specific *Vibrio* species, with production occurring in two strains isolated from very different geographical regions and sources.

The antibacterial compound of *P. halotolerans* S2753 was identified as holomycin (Rt = 1.70 min; Figure 2), a compound belonging to the pyrrothine class of antibiotics acting by interference with RNA synthesis [52]. Our NMR data (Figure 3b) was consistent with previous reports [53]. Holomycin has until now only been found in Gram-positive *Streptomyces* [54,55], and the present study is the first demonstrating production of this antibiotic in a Gram-negative heterotrophic bacterium. While parallel evolution of this trait is possible, horizontal gene transfer is the more likely explanation for its occurrence in both *Vibrionaceae* and actinomycetes. We extended the broad-spectrum activity of holomycin [52] by showing inhibition of the bacterial pathogens *Listeria monocytogenes*, *Serratia marcescens*, *S.* Enteritidis, *B. cereus*, *Y. enterolitica*, *Y. ruckeri*, *V. harveyi*, *V. vulnificus* and *V. parahaemolyticus*, as well as of several marine strains from the *Roseobacter* and *Pseudoalteromonas* groups (data not shown).

Neither andrimid nor holomycin were produced by *V. neptunius* S2394 and S4051, and further fractionation and purification is needed to identify the compound(s) responsible for their antibacterial activity. Interestingly, *V. neptunius* S4051 and *V. coralliilyticus* S4053 were isolated from the same seaweed sample, showing that two antagonistic *Vibrio* species whose antibacterial activity is based on different compounds co-occur in the same microenvironment. Moreover, the same sample also contained an antibiotic-producing *Pseudoalteromonas* strain [8].

Two of the antagonistic *Vibrionaceae* species harbor pathogenic strains, with *V. coralliilyticus* being pathogenic to corals [56] and *V. neptunius* being pathogenic to oysters [57]. While we do not know whether *V. coralliilyticus* S2052 has pathogenic potential, the *V. coralliilyticus* type strain has both antagonistic and pathogenic traits [12]. Hence, our results suggest that some vibrios possess a dual physiology, being antagonistic against other prokaryotes but pathogenic towards higher organisms. Moreover, the production of antibiotics in several species suggests that these compounds may be of ecological importance [1].

This study highlights one of the challenges in natural product discovery. Despite major screening efforts for novel antimicrobials to be used in pharmaceutical, food, and aquaculture industries, only a limited amount of compounds have been discovered in recent years [58]. While the isolation of culturable bacteria remains a promising approach [42] and the secondary metabolome of marine vibrios has not been extensively studied, we only isolated known compounds despite careful dereplication prior to any compound purification. Dereplication is apparently troubled by the high degree of gene transfer between distantly related bacteria such as Gram-positive actinomycetes and Gram-negative *Proteobacteria* [3]. Many compounds in natural product databases such as AntiBase [59] have similar masses (<5 ppm difference), so even the combination of UV/VIS spectra, accurate mass data (<5 ppm), and E-SPE [45] is not sufficiently discriminatory for these organisms. To avoid isolation of redundant chemistry, dereplication by NMR [60] or ultra high-resolution mass spectrometry (<1 ppm) with high isotope accuracy ratios for correct elementary composition determination [61] is imperative to exclude previously isolated compounds.

## 3. Experimental Section

### 3.1. Isolation of Bioactive Marine Vibrionaceae

During a global research expedition (http://www.galathea3.dk/uk), marine bacterial strains were isolated from environmental samples and screened for antagonistic activity against a pathogenic *Vibrio anguillarum*, strain 90-11-287. Three hundred and one bioactive strains were identified as *Vibrionaceae* based on 16S rRNA gene similarities [8]. Pure cultures of strains were stored in cryoprotectant solution at −80 °C until being analyzed in the present study.

### 3.2. Selection of Strains with Pronounced Antibacterial Activity

All 301 strains were retested for antibacterial activity by spotting colony mass on agar seeded with either *V. anguillarum* strain 90-11-287 or *S. aureus* strain 8325. Activity was assessed by the formation of clearing zones around spotted colony mass. Selected active strains were grown both stagnant and aerated (200 rpm) in 30 mL Marine Broth 2216 (Difco 279110) for 3 days at 25 °C in 250 mL glass bottles. Cultures were extracted with an equal volume of HPLC-grade ethyl acetate (EtOAc) for 30 min. The organic phase was transferred to fresh sample vials and evaporated under nitrogen until dryness. Extracts were redissolved in 1 mL of EtOAc and stored at −20 °C until further analysis. EtOAc extracts were tested in a well diffusion agar assay [62] for activity against *V. anguillarum* strain 90-11-287 and *S. aureus* strain 8325.

### 3.3. Phylogenetic Analysis

Genomic DNA was extracted from 1-day cultures using the NucleoSpin Tissue Kit (Macherey-Nagel, Düren, Germany) according to the manufacturer’s instructions. PCR for *recA* and *toxR* gene fragments was performed according to [35], and PCR for *rpoA* gene fragments according to [34]. PCR products were checked by agarose gel electrophoresis and purified using the Wizard PCR Preps DNA Direct Purification System (Promega, Madison, USA) according to the manufacturer’s instructions. Obtained nucleotide sequences were edited using Chromas Lite (Technelysium, Australia) and aligned to its closest sequence relative [36]. The phylogenetic relationship between the five isolates was determined by neighbor-joining analyses (1000 bootstrap replicates) of nucleotide and amino acid alignments (translated using EMBOSS Transeq, http://www.ebi.ac.uk/Tools/emboss/transeq/) done in ClustalX. Gene sequences have been deposited at GenBank under the accession numbers HQ452614–452618 (*toxR*), HQ452619–452623 (*recA*), and HQ452624–452628 (*rpoA*).

### 3.4. Influence of Culture Conditions on Bioactivity

The five strains with strongest antibacterial activity (S2052, S2394, S2753, S4051, and S4053) were grown both stagnant and aerated (200 rpm) at 25 °C in either Marine Broth (MB) or Marine Minimal Medium [63] containing 0.4% glucose and 0.3% casamino acids (MMM). Per strain and culture condition, three bottles were inoculated with 30 mL of medium each, of which each one was sampled after 1, 3, and 5 days of incubation. In addition, strains were grown in 30 mL sea salt solution (Sigma S9883; 40 g L^−1^) with 0.4% glucose and 0.3% casamino acids for 3 days (200 rpm) at 25 °C. EtOAc extracts were prepared as described above, and tested in a well diffusion agar assay [62] for activity against *V. anguillarum* strain 90-11-287 and *S. aureus* strain 8325.

### 3.5. Chemotyping

Liquid chromatography-diode array/mass spectrometry (LC-UV/MS) analyses were performed on dried EtOAc extracts redissolved in methanol (MeOH) from all tested culture conditions to visualize the array of produced molecules. In addition, 3-day MMM cultures were extracted and analyzed in biological triplicate. LC-UV/MS was performed on an Agilent 1100 liquid chromatograph with a diode array detector (Agilent, Waldbronn, Germany) coupled to an LCT TOF mass spectrometer (Micromass, Manchester, UK) using a Z-spray ESI source. The separation was done on a Luna II C_18_ column (50 mm × 2 mm, 3 μm) (Phenomenex, Torrance, CA) fitted with a security guard system using a linear gradient starting from 15% acetonitrile (MeCN) in water (H_2_O) to 100% MeCN over 20 min at a flow rate of 300 μL min^−1^. Both MeCN (HPLC grade) and H_2_O were buffered with 20 mM HPLC-grade formic acid (FA).

### 3.6. Isolation and Structural Elucidation of Antibacterial Compounds

Strains S2052 and S2753 were grown in 20 L sea salt solution (Sigma S9883; 40 g L^−1^) with 0.4% glucose and 0.3% casamino acids for 3 days (100 rpm) at 25 °C. On day 3, sterile Dianion HP20SS resin (Sigma-Aldrich, St. Louis, MO) was added to the broth (12 g of resin L^−1^). After 24 h, the resin was filtered off and washed with H_2_O (2 × 1 L), followed by extraction with MeCN/H_2_O (80/20 v/v; 2 × 1500 mL).

For S2052, all organic extracts were pooled, absorbed onto 90 g Sepra ZT C18 (Phenomenex), and dried before packing into a 100 g SNAP column (Biotage, Uppsala, Sweden) with pure resin (10 g) in the base. Using an Isolera flash purification system (Biotage), the extract was subjected to a crude fractionation using a MeCN/H_2_O gradient (flow rate 30 mL min^−1^) starting with 10% MeCN (10 min, isocratic), increasing to 100% MeCN (25 min) before washing with 100% MeCN (15 min). Fractions were automatically collected using UV detection (210 and 320 nm). The fraction with antibacterial activity (185 mg) was subjected to further purification on a Luna II C_18_ column (250 × 10 mm, 5 μm) (Phenomenex) using a 45–70% MeCN/H_2_O gradient (buffered with 20 mM FA, flow rate 5 mL min^−1^) over 20 minutes on a Gilson 322 liquid chromatograph with a 215 liquid handler/injector (BioLab, Risskov, Denmark). This yielded 7.6 mg of pure andrimid.

For S2753, the MeCN/H_2_O extract from Dianion HP20SS extraction was evaporated until dryness on a rotary evaporator. The extract was redissolved in EtOAc, absorbed onto 5 g Isolute diol (Biotage), and added to a glass column with pure diol (95 g). A total of 12 fractions were collected from the diol column (100 g, 20 × 350 mm) ranging from heptane, dichloromethane, EtOAc to pure MeOH, running under gravity. The fraction with antibacterial activity (172 mg, 100% EtOAc) was further separated on the Isolera flash purification system, on Sepra ZT C18 (10 g SNAP) using a MeCN/H_2_O gradient (flow rate 12 mL min^−1^) starting with 5% MeCN increasing to 30% MeCN (12 min), quickly increasing to 100% MeCN (10 min). Fractions were automatically collected using UV detection (210 and 380 nm). Pure holomycin (4.3 mg) was obtained after final purification on a Luna II C_18_ column (250 × 10 mm, 5 μm) (Phenomenex) using a MeCN/H_2_O (buffered with 20 mM FA) gradient from 7–37% MeCN over 17 min.

NMR spectra were recorded on a Bruker Avance 800 MHz spectrometer with a 5 mm TCI Cryoprobe at the Danish Instrument Center for NMR Spectroscopy of Biological Macromolecules, using standard pulse sequences. The NMR data used for the structural assignment of andrimid and holomycin were acquired in DMSO-*d_6_* (δ_H_ 2.49 and *δ*_C_ 39.5 ppm).

Optical rotation was measured on a Perkin Elmer Model 341 polarimeter (Perkin Elmer, Waltham, MA) (α_D_ at 589 nm).

#### Andrimid

orange-yellow amorphous solid; UV (MeCN/H_2_O) λ_max_ 200 (100%), 280 (40%) nm; [α]*_D_*^20^ −62.9° (*c* 0.24, MeOH); ^1^H NMR δ_H_ ppm: 0.75 (3H, d, 6.7 Hz, H-4′), 0.80 (3H, d, 6.7 Hz, H-5′), 1.07 (d, 7.2 Hz, 1H, H-6), 1.78 (3H, d, 6.7 Hz, H-8‴), 2.30 (m, 1H, H-3′), 2.65 (1H, dd, 14.6, 6.2 Hz, H-2_a_″), 2.77 (1H, dd, 14.6, 8.2 Hz, H-2_b_″), 2.91 (m, 1H, H-4), 3.92 (d, 5.6 Hz, 1H, H-3), 4.62 (dd, 8.4, 5.4 Hz, 1H, H-2′), 5.28 (1H, m, H-3″), 5.90 (1H, m, H-7‴), 6.01 (1H, d, 15.2 Hz, H-2‴), 6.18 (1H, m, H-6‴), 6.26 (1H, dd, 14.5, 11.4 Hz, H-4‴), 6.53 (1H, dd, 14.5, 10.0 Hz, H-5‴), 7.00 (1H, dd, 15.2, 11.4 Hz, H-3‴), 7.20 (1H, m, H-7″), 7.29-7.31 (4H, m, H-5″/H-6″), 8.11 (1H, d, 8.4 Hz, NH-2′), 8.42 (1H, d, 8.5 Hz, NH-3″), 11.36 ( s, 1H, NH-1); ^13^C NMR δ_C_ ppm: 14.5 (C-6), 17.2 (C-4′), 18.3 (C-8‴), 19.4 (C-5′), 28.1 (C-3′), 39.0 (C-4), 41.9 (C-2″), 57.8 (C-3), 63.1 (C-2′), 124.2 (C-2‴), 126.4 (C-5″), 126.9 (C-7″), 128.1 (C-4‴), 128.2 (C-6″), 131.5 (C-6‴), 133.4 (C-7‴), 139.0 (C-5‴), 139.4 (C-3‴), 142.9 (C-4″), 164.3 (C-1‴), 169.9 (C-1″), 173.8 (C-2), 180.0 (C-5), 203.9 (C-1′); HRESIMS *m/z* 479.2435 (calcd for C_27_H_33_N_3_O_5_, 479.2420).

#### Holomycin

orange-yellow prisms; UV (MeCN/H_2_O) λ_max_ 200 (100%), 280 (40%) nm; ^1^H NMR δ_H_ ppm: 2.01 (s, 1H, H-9), 7.04 (s, 1H, H-1), 9.86 (s, 1H, NH-7), 10.69 (s, 1H, NH-3); ^13^C NMR δ_C_ ppm: 22.4 (C9), 110.6 (C-1), 115.4 (C-5), 133.7 (C-2), 133.9 (C-6), 167.9 (C-4), 168.8 (C-8); HRESIMS *m/z* 213.9860 (calcd for C_7_H_6_N_2_O_2_S_2_, 213.9871).

## 4. Conclusions

The present study adds to the knowledge of *Vibrionaceae* bioactivity and physiology by showing a worldwide occurrence of marine strains producing antibacterial compounds. In addition, we underlined that chemotyping can support gene-based species identification and help resolving phylogenetic relationships within a genetically homogenous family such as the *Vibrionaceae*. The discovery of known antibiotics that are also produced by evolutionary distant microbes suggests an involvement of horizontal gene transfer, and indicates that these compounds are fundamental to compete and communicate in the natural habitat. The cosmopolitanism of identical antibiotics has major implications for natural product discovery strategies and stresses the need for careful dereplication in the initial stages of screening. An alternative approach could be the screening for largely untested bioactivities, for instance, interference with quorum sensing or modulation of gene expression.

## Figures and Tables

**Table 1:** Inhibition of *V. anguillarum* strain 90-11-287 and *S. aureus* strain 8325 by ethyl acetate extracts from five marine *Vibrionaceae*. Antibacterial activity is displayed by the diameter of clearing zones (–: no activity; +: between 0 and 15 mm; ++: between 15 and 30 mm; +++: over 30 mm).

		Inhibition of	
Strain	Species	*V. anguillarum*	*S. aureus*
S2052	*V. coralliilyticus*	+++	++
S2394	*V. neptunius*	++	−
S2753	*P. halotolerans*	+++	++
S4051	*V. neptunius*	++	−
S4053	*V. coralliilyticus*	++	+
